# Do genetics contribute to TNF inhibitor response prediction in Psoriatic Arthritis?

**DOI:** 10.1038/s41397-022-00290-8

**Published:** 2022-10-15

**Authors:** Philippa D. K. Curry, Andrew P. Morris, Anne Barton, James Bluett

**Affiliations:** 1 Versus Arthritis Centre for Genetics and Genomics, Centre for Musculoskeletal Research, The University of Manchester, Manchester, UK; 2 NIHR Manchester Biomedical Research Centre, Central Manchester NHS Foundation Trust, Manchester Academic Health Science Centre, Manchester, UK

**Keywords:** Predictive markers, Genetic markers, Rheumatic diseases

## Abstract

Psoriatic arthritis (PsA) is a heterogeneous chronic musculoskeletal disease, affecting up to 30% of people with psoriasis. Research into PsA pathogenesis has led to the development of targeted therapies, including Tumor Necrosis Factor inhibitors (TNF-i). Good response is only achieved by ~60% of patients leading to ‘trial and error’ drug management approaches, adverse reactions and increasing healthcare costs. Robust and well-validated biomarker identification, and subsequent development of sensitive and specific assays, would facilitate the implementation of a stratified approach into clinical care. This review will summarise potential genetic biomarkers for TNF-i (adalimumab, etanercept and infliximab) response that have been reported to date. It will also comment upon the importance of managing clinical confounders when understanding drug response prediction. Variants in multiple gene regions including *TNF-A, FCGR2A, TNFAIP3, TNFR1/TNFR1A/TNFRSF1A, TRAIL-R1/TNFRSF10A, FCGR3A* have been reported to correlate with TNF-i response at various levels of statistical significance in patients with PsA. However, results were often from heterogenous and underpowered cohorts and none are currently implemented into clinical practice. External validation of genetic biomarkers in large, well-documented cohorts is required, and assessment of the predictive value of combining multiple genetic biomarkers with clinical measures is essential to clinically embed pharmacogenomics into PsA drug management.

## Psoriatic arthritis

A chronic inflammatory joint disease, psoriatic arthritis (PsA) has a prevalence of up to 30% in patients with psoriasis (Ps) [1, 2]. It is a heterogenous and multisystem condition with varied symptom severity and localisation, and the disease course is often unpredictable [3, 4]. Phenotypes include painful swelling, stiffness, nail disease, dactylitis and enthesitis, affecting both the peripheral joints and axial skeleton, with 47% of patients demonstrating bone erosion within 2 years of diagnosis [5–9]. Beyond musculoskeletal and dermatological symptoms, PsA patients also experience fatigue, reduced social participation, sleeping difficulties and diminished work capacity with one study finding 39% of PsA patients were work-disabled [10, 11]. Furthermore, over half of PsA patients have at least one comorbidity with an increased level of mortality risk, including cardiovascular diseases being linked to 36% of deaths [5, 7, 12–18].

## Tumour Necrosis Factor inhibitors

As the knowledge of psoriatic immunopathogenesis improved over the past 20 years, the therapeutic advancement of biologic drugs has also expanded dramatically. Drugs targeting Tumour Necrosis Factor (TNF) and IL-12/23/17 pathways, key in disease pathophysiology, have been developed [19]. TNF is a pleiotropic pro-inflammatory cytokine whose over-expression has been identified as one of the major downstream effectors in PsA development. With expanded production triggered by macrophages, neutrophils and T-cells, TNF works as a key activator, resulting in high levels of cytokines, activated macrophages, monocytes, fibroblasts, mast and endothelial cells via the Th1 and Th17 pathways, overall emphasising the increased inflammatory state of PsA [20, 21]. Enhanced TNF also contributes to increased cardiovascular risk, angiogenesis, proliferation, and apoptosis. Drugs targeting TNF are currently the frontline European League Against Rheumatism (EULAR) treatment choice of targeted biologic therapy for severe and chronic PsA patients. This is due to their effectiveness at targeting and inhibiting progression in multiple aspects of the heterogenous condition simultaneously, including pain, psoriasis, enthesitis, fatigue and slowing progressive and debilitating bone erosion commonly seen in PsA [21]. This, and their long-term safety and efficacy, makes them an appealing and preferred therapeutic option for patients with heterogenous phenotypes.

There are currently five TNF-inhibitor (TNF-i) drugs available for the management of PsA that have been approved by the Food and Drug Administration. Two classes of drug exist: TNF targeting monoclonal antibodies (adalimumab (ADA), infliximab (IFX), golimumab, and certolizumab pegol) and TNF fusion protein receptor inhibitors (etanercept (ETN)). Additionally, ADA, IFX and golimumab are full-length bivalent monoclonal antibodies while certolizumab pegol is a humanized IgG1 monoclonal antibody with a Fab’ fragment conjugated to polyethylene glycol. ETN is a genetically modified soluble fusion protein made of two extracellular portions of p75 TNFR linked to human IgG1 Fc [21]. Both classes are associated with higher levels of response when compared to placebo, in both musculoskeletal and dermatological symptoms, as well as protection for bone erosion and destruction in both the axial skeleton and peripheral joints [22, 23]. Despite ETN becoming the first TNF-i to demonstrate a significant response in PsA, ADA is the most commonly prescribed TNF-i, accounting for 40% of prescriptions in some PsA cohorts [8, 24]. TNF-i are more expensive than conventional disease modifying anti-rheumatic drug alternatives, including Methotrexate (MTX) and Sulfasalazine, although increased costs are offset by significantly improved efficacy [25]. However, lack of drug adherence and indirect disease costs are also reported to add to the economic burden of PsA [26]. Furthermore, in approximately 40% of patients, the first biologic drug is ineffective, with significantly higher rates of non-response for patients receiving a second TNF-i agent [5, 27, 28]. This highlights the need for the identification and validation of robust biomarkers of response to achieve a stratified medicine approach, whereby drugs can be targeted to the patients most likely to benefit from them.

PsA is a multifactorial disease with lifestyle factors such as Body Mass Index, exercise and smoking associated with risk of PsA development [5]. Nevertheless, there is a robust evidence base to support a genetic component to PsA disease susceptibility with high heritability reported (*h*^2^ = 0.41) (ref. [4, 27]). Heritability is a measure of the contribution of genetics to a displayed phenotype. Here, over 40% of PsA development is thought to be attributable to genetic variation. Importantly, despite the strong evidence the role of genetics in PsA, genetic variation may also influence response to therapy. Thus far, few studies have investigated the extent to which genetics contributes to treatment response. To successfully implement pharmacogenomics, and a personalised approach to PsA treatment, an expanded understanding of the role of all factors associated with response is imperative. This review will evaluate the role of genetic biomarkers and clinical factors that have been reported to associate with TNF-i response in PsA.

## Clinical and lifestyle factors correlated with TNF-i response in PsA

Predicting which patients respond to a drug will save time, money, and debilitation in a complex condition, such as PsA, where there are multiple influencers of treatment response. To assess the utility of genetic predictors of response, all other clinical predictors also need to be acknowledged and understood.

Female sex has been negatively associated with drug persistence and EULAR response in PsA cohorts across multiple TNF-i drugs and is considered the most consistent predictor of response [11, 29–33]. A correlation between increased testosterone levels and HLA-B27 presence in spondylarthritis diseases and higher levels of concomitant generalized pain syndrome in females have been postulated to contribute to the differences observed [34–36]. Age at therapy initiation showed no effect on drug persistence [11, 37, 38]. The presence of comorbidities, assessed using the Charlson comorbidity index, found that Danish PsA patients (*n* = 1750) with Charlson comorbidity index >2 had higher baseline disease activity, increased occurrence of depression and anxiety and shorter TNF-i persistence compared to Charlson comorbidity index = 0 [39]. Anxiety or depression may impact drug response due to the pro-inflammatory cytokines and altered immune response observed with psychiatric conditions [40]. However, a direct causal effect has not yet been established in inflammatory diseases. Comorbidities also influence TNF-i therapy choice, with ETN advised for those with high infection or cardiovascular risk [41]. Lifestyle factors have also been reported to correlate with TNF-i response, with current smoking and higher alcohol intake having a negative correlation with drug persistence and 6 month EULAR response respectively [11, 36, 42]. Obesity is associated with cytokine production and higher levels of inflammation; it has also been consistently negatively correlated with response to TNF-i in patients with PsA patients [36, 39, 43–45]. In a systematic meta-analysis review of patients with autoimmune diseases (*n* = 19,372), almost a quarter of all Ps and PsA patients (23% of *n* = 11,873) were obese, and TNF-i were less likely to be effective in obese patients [46]. Overall, a multi-disciplinary approach to treatment is recommended, targeting weight reduction in obese patients in parallel to TNF-i drug therapy [46–48]. Duration of PsA disease prior to commencing TNF-i treatment shows conflicting evidence for association with response to therapy [37, 38, 49, 50]. Whilst lower skin involvement (<20 on the global physician skin assessment) has been associated with increased drug persistence [49], one study found PsA disease distribution did not influence overall persistence rates, although those with prevalent axial involvement did have longer durations on their initial biologic [29]. Individuals with lower baseline disease activity, including lower tender joint count, have been reported to have improved drug persistence and EULAR response in PsA [36, 49, 50]. However, baseline Health Assessment Questionnaire, a measure of disability, has not been associated with treatment response, unlike in rheumatoid arthritis (RA) [38].

Choice of therapy may influence drug response, although results remain inconclusive. Some studies report no differences in persistence rates between first-line biologics [11]; one found increased persistence with ADA. Both Finnish and British cohorts have reported significantly lower persistence for IFX whilst, by contrast, a Greek study found that IFX had the best persistence rate in the first 6 months, with ADA reported to have the highest prescription rates after that time point [24, 29, 31, 42]. Response levels and persistence levels decline for second-line biologics following primary failure, a figure that declines further with subsequent therapies when compared to PsA biologic naïve patients [49, 50]. A PsA cohort (*n* = 765) reported a decline in efficacy from first to third biologic (68% vs. 37% moderate EULAR response), although conflicting results still remain [24, 37, 41]. Another clinical factor that has conflicting results on therapeutic response is co-therapy with conventional synthetic Disease Modifying Antirheumatic Drugs, particularly MTX, with IFX reported as having the highest level of co-therapy [11]. PsA patients on concomitant treatment with glucocorticoids or MTX are reported to have higher levels of biologic persistence and response in some studies [11, 29, 30, 36, 39, 42, 51], but not others [24, 29, 38, 49, 52].

The development of anti-drug antibodies (immunogenicity) has been associated with reduced efficacy to monoclonal TNF-i therapy in PsA [53–55]. In a well-characterised PsA cohort, ADA levels were significantly associated with increased levels of Anti-Drug Antibodies (ADAb), a reduction in Health Assessment Questionnaire scores, and were positively correlated with Disease Activity Score 28. Additionally, lower levels of ADAb were seen in patients on concomitant MTX therapy compared to ADA alone (~6% vs. ~72%). In comparison, no association was seen in ETN-treated patients with no patients having developed detectable ADAb [53]. In a more heterogenous cohort, including 58 TNF-i Chinese patients (PsA *n* = 10), the development of ADAb was reported in 50% of IFX patients, 31% of ADA and none in ETN patients. Patients with anti-drug antibodies were more likely to be classified as ‘non-responders’, have lower drug levels and higher rates of drug inefficacy and withdrawal. Combination therapy with MTX showed a protective effect, while female sex was associated with higher levels of antibodies to TNF-i monotherapy; this supports the idea of females experiencing higher levels of immunogenicity [56]. TNF-i therapy adherence levels in chronic inflammatory diseases, including PsA, have been reported as suboptimal (59%), with adverse reactions, forgetting, high comorbidity and disease burden, and medication concerns being listed as reasons for non-adherence in RA patients [57, 58]. Furthermore, poor adherence was significantly associated with decreased TNF-i treatment response in RA, however, further work, in well-defined PsA cohorts is needed [59].

Overall, understanding the contribution of clinical factors and their association with drug response is imperative. This will allow the appropriate and personalised management of some of these factors, including adherence, comorbidities, and mental health conditions. Furthermore, it is realistic that the development of any prediction models of pharmacogenetic drug response will include well validated and identified clinical markers of response.

## Genetic biomarkers of response

Due to the highly varied interindividual biologic efficacy and response to treatment, there is great potential to improve the management of PsA patients therapy via the identification of predictive biomarkers [60]. DNA variants, typically single nucleotide polymorphisms (SNPs), can make ideal biomarker candidates in PsA owing to their fixed nature, availability prior to commencing treatment, and the reducing costs of genotyping [22]. Some variants identified are also specific for certain treatments, whereas others are reported with more of a class effect. If genetic variants that correlate with response to TNF-i drugs in patients with PsA could be identified and robustly validated, it would pave the way for a stratified medicine approach in clinics [61]. Furthermore, genetic risk scores, created from aggregating information from multiple genetic variants simultaneously could increase predictive power further [62]. Thus far, no pharmacogenomic markers of PsA development or response are in place clinically. There is strong evidence for the role of the HLA region in PsA development, however, due to the modest effect of variants in this region on overall risk and limited positive and negative predictive values, no test is routinely used [20]. By gaining an enhanced understanding of the role of pharmacogenomics in PsA development and drug response, via large well documented and validated studies, and by combining this information in risk score analysis it could improve the presence of pharmacogenomics in clinics, aiding the prediction of TNF-i response. The development of economically and rapid clinical tests with high positive and negative predictive values is also imperative.

## Adalimumab

ADA is a TNF targeting monoclonal antibody and is prescribed as an immunosuppressant for many autoimmune diseases. Several studies have investigated genetic variants in the TNF-alpha gene (*TNF-a*) for association with response to ADA, given that the gene is the target of the drug. Part of the major histocompatibility complex class III region on the shorter arm of chromosome 6, the gene is located between *HLA-B* and *HLA-DR* [63, 64]. Interestingly, in a small study (*n* = 57) evaluating the role of *TNF-a* polymorphisms with different TNF-i response, a variant in the first intron of the gene was associated with both first- or second-line ADA responders [63]. Responders all achieved either American College of Rheumatology 70 or American College of Rheumatology 70 by 6 months as well as an improvement in Disease Activity Score 28 value (>1.2) at the same time period, with some achieving both measures by 3 months. Out of the 16 patients on ADA, the *TNF-a* + 489 A/A genotype (rs80267959) was observed in responders (*n* = 5/13) but not in non-responders (*n* = 0/3) (Table 1). In comparison, no association was reported in either IFX or ETN. Although the cohort is very small, the reported association merits further investigation in larger replication studies [63].

**Table 1:** Polymorphisms reported to influence TNF-i response.

Drug	Gene	Rs ID	Cohort	Reference
ADA	*TNF-A*	rs80267959	57 PsA	[64]
ETN	*TNF-A*	rs80267959	57 PsA	[64]
		rs1800629	86 (54 RA, 10 PsA, 22 AS)	[66]
ETN	*FCGR2A*	rs1801274	103 PsA	[71]
ETN	*TNFAIP3*	rs610604 rs6920220	20 Psa and Ps	[69]
IFX	*TNFR1/ TNFR1A*	rs767455	145 (90 RA, 55 PsA)	[74]
	*/TNFRSF1A*	rs1800693	137 (82 PsA, 55 AS)	[68]
IFX	*TRAIL-R1/ TNFRSF10A*	rs20575	145 (90 RA, 55 PsA)	[41]
IFX	*FCGR3A*	rs36991	90 (41 RA, 16 PsA, 33 AS)	[72]

## Etanercept

ETN is a dimeric fusion protein that acts as a TNF-α receptor inactivating TNF following binding. Due to its role in PsA aetiopathogenesis *TNF-a* polymorphisms were amongst the first studied to determine their influence of drug response variability. Multiple polymorphisms in the *TNF-a* promotor region have been investigated for their role in PsA development but also specifically in ETN response including −308 (rs1800629), +489 (rs80267959) and −238 (rs361525), with the first polymorphism to be associated with PsA drug response identified at position −308 (Table 1) (ref. [62]). In a mixed inflammatory arthritis cohort (*n* = 86) the −308 G/G genotype was associated with increased response to ETN independently, as well as in combination with other TNF-i (ADA and IFX) [65]. Under an additive genetic model, patients carrying G alleles displayed an improved response to all TNF-i drugs, regardless of the underlying disease. The best results were observed in patients with the G/G genotype, with a ‘good response’ exclusively seen in this cohort. In contrast, 90% of A/G patients also achieved a ‘moderate response’, while all patients with A/A genotypes failed to respond [65]. Of note, all PsA patients carried the G/G genotype and were good responders. This is supported by similar results in a Ps cohort, including PsA patients [66]. However, the result has not been replicated in any independent studies, including one undertaken in an Italian population [61, 63, 67]. In a separate cohort of patients with PsA (*n* = 57), the  + 489 *TNF-a* promotor was reported to not only be associated with PsA disease severity and development, but also with ETN response [63]. *TNF-a* + 489 G/G and G/A genotypes were observed at an increased frequency in responders compared to non-responders, while all A/A patients demonstrated no response, although this finding failed to reach statistical significance. In contrast, association was noted with the −238 variant (rs361525) and ETN response. Further lack of evidence for the influence of −238 and −308 polymorphisms on TNF-i response has also been reported [63, 68]. The importance of investigating the combined impact of haplotypes on TNF-i response, rather than just single nucleotide polymorphisms, has also been highlighted [63, 69]. Although the results are non-significant, given that the gene is the drug target, these polymorphisms should be explored in larger cohorts to confirm the results [63]. Studies to date, have included a limited number of PsA patients (*n* = 10–103) which impacts power to detect an association.

The fragment crystallizable region of TNF-i drugs, including ETN, binds to Fc-y receptors and so the effect of polymorphisms in genes influencing the affinity to this region, *FCGR2A-131* and *FCGR3A*, on response has been investigated. In PsA patients (*n* = 103) prescribed ETN (~53%), IFX or ADA, higher affinity alleles, in either the homozygous or heterozygous state, variants in *FCGR2A-131* (rs1801274) associated with a higher 6 month response [70]. Following stratification based on prescribed TNF-i, the effect was greatest in patients on ETN, with an association with response also seen by 3 months. In contrast, no *FCGR3A* polymorphisms showed an association in any drug, which is supported by other PsA studies [71]. Interestingly, the *FCGR2A-131* high affinity findings contradict those in RA where low affinity alleles are linked to a better response to IFX therapy [72].

Furthermore, when investigating the effect of TNF-i response collectively, a modest Spanish PsA cohort (*n* = 20) reported that rs610604 and rs6920220 in *TNFAIP3* are significantly associated with quality of life improvement at both the 3 and 6-month stages [68]. This is in accordance with previous Ps pharmacogenetic studies and demonstrates a promising potential response marker for TNF-i. Further work is needed to confirm these associations, in larger cohort studies with well-described outcome measures.

## Infliximab

A chimeric monoclonal antibody, IFX binds and neutralizes TNF-a. Polymorphisms associated with response to IFX have been reported in multiple genes (*TNF-R1A/TRAIL1/FCGR3A*), with various levels of validation in different cohorts (Table 1). Tumour Necrosis Factor-related Apoptosis-inducing Ligand Receptor 1 (*TRAILR1)* and Tumour Necrosis Factor Receptor 1 A (*TNF-R1A/TNF-R1/TNFRSF1A*) have been associated with both chronic inflammatory diseases and cancer susceptibility, thus these death receptors are thought to play an important role in immune system homeostasis. *TNF-R1A* rs767455 was associated with a significantly improved 3-month EULAR response in 55 PsA patients with the homozygous A/A genotype versus the homozygous G/G or heterozygous A/G genotype, with the A allele significantly associated with a better response. Of interest, this demonstrated an opposing effect to that seen at 3 months in RA patients, potentially highlighting a distinct difference in pathophysiology between the diseases [61, 73]. In a separate Italian spondyloarthritis cohort of 65 patients (PsA=59%) undergoing TNF-i therapy, the role of the TNF-TNFR pathway in TNF-i response was investigated. The TNF receptor superfamily member 1A (*TNFRSF1A*) rs1800693, rare G/G genotype, was associated with delayed response to IFX but not ADA therapy. Response was assessed using The Bath Ankylosing Spondylitis Disease Activity Index [67]. The presence of this polymorphism is thought to produce a truncated TNFR-1 protein and disruption of the TNF-a/TNFR1 balance [74]. The potential confounding effect of physiological, pathophysiological and environmental factors was noted and the importance of investigating their influence highlighted [67]. *TRAILR1/TNFRSF10A* (8p21) is part of the TNF-receptor superfamily and is involved in cell apoptosis induction. The association of *TRAILR1* polymorphisms was investigated in a mixed cohort of RA and PsA. In a subgroup analysis of 27 IFX-treated PsA patients, patients with rs20575 C/C genotype in *TRAILR1* demonstrated an improved 6 month EULAR response to IFX [73]. *FCGR3A* encodes for the fragment crystallizable portion of immunoglobulin G and is a mediator of antibody cell mediated cytotoxicity. In one study rs396991 V158F higher affinity V/F and V/V genotypes were associated with a significantly improved 3 month EULAR response in PsA (*n* = 16) patients, perhaps due to increased immunoglobin binding and natural killer activation, although the small cohort size is of note [71, 75]. In contrast, RA patients had an increased response with F/F genotypes [71], although contradictory results of the influence of this polymorphism have been reported [70]. These results demonstrate the difference, not only between autoimmune diseases but also between TNF-i. However, a lack of consistency between chosen response criteria selected by studies is observed. Despite this, some genes involved in PsA pathophysiology have been identified to harbour promising candidate polymorphisms for TNF-i response prediction (*TRAIL-R1/ TNFRSF10A, FCGR3A*).

## Golimumab and certolizumab pegol

Currently, no validated associations between genetic biomarkers and response to golimumab or certolizumab pegol have been identified, probably due to the lower frequency of prescription of these drugs. However, in 20 PsA patients treated with TNF-is, transcriptomic profiling of immune cells at baseline and after 3 months identified differentially expressed genes between responders and non-responders at the 3 month stage, highlighting key novel pathways and genes involved with response, including the JAK and STAT pathways. Clustering via principal component analysis based on cell-type-specific transcriptomic data at baseline was able to cluster responders and non-responders for different treatments, suggesting that further investigation is needed to determine whether it is a prediction marker for response [76].

## Conclusions

The future of stratified medicine requires the identification of robust and validated biomarker panels. Genetic variants are attractive as potential biomarkers as they only need to be tested once; furthermore, in other areas of medicine, genetic variants have been reported to associate with treatment effects [61, 77, 78]. Whilst several candidate genetic polymorphisms have been discussed in this review, few have been subsequently replicated or have a large enough effect size to be translated to the clinic. Notably, investigations have been conducted using modest and underpowered candidate gene studies with high levels of heterogeneity, particularly in recruited disease and response measurements. Indeed, a significant limitation to the detection of response biomarkers is statistical power and a lack of appropriate adjustment for clinical variables such as adherence. The largest cohort identified in the literature review recruited 103 PsA patients, which is significantly lower than pharmacogenetic studies of TNF-i response in RA (*n* >1 500) ([77]). Overall, this has restricted the amount of work that can be conducted to start to predict TNF-i response in PsA and include genetics into these models. To date, no successful models of TNF-i prediction in PsA are available clinically.

PsA is a clinically heterogeneous disease, affecting the skin, axial and peripheral skeleton. Whilst several outcome measures exist, the majority have been developed initially in RA, a disease that is genetically distinct from PsA. In addition, most outcome measures used in PsA studies include a subjective assessment of response which may be affected by other diseases such as depression or chronic non-inflammatory pain. The lack of specific and objective PsA outcome measures for pharmacogenetic studies is a significant barrier to the identification of genetic biomarkers of response and should be an area for future research. Furthermore, future work should also focus on the association between genetic variants and drug response in specific disease domains, rather than non-representative disease activity composite scores. By investigating response in enthesitis, psoriasis or joint erosion domains, independently, the role of pharmacogenomics in PsA response could be elucidated, whilst reducing heterogeneity in PsA and enhancing opportunities for personalised medicine approaches.

Whilst several clinical factors are associated with TNF-i response, none are of significant effect size to be used alone and it is likely that they will need to be combined with other biomarkers of response to make clinical decisions. To date, few pharmacogenetic studies have included clinical factors, such as adherence, in the identification of genetic biomarkers of response, perhaps due to the small sample size available.

For these reasons, there are currently no validated clinically used genetic biomarkers of response in PsA. To successfully include genetic biomarkers in the development of predictive models of TNF-i response, there remains a need for hypothesis-free genome-wide association studies in large well-characterised cohorts where response to treatment has been consistently measured. Due to the prevalence of PsA, it is likely that such cohorts would need to be developed through international collaboration to improve sample size and power. Furthermore, the role of potential confounders of response, including ADAb, adherence, concomitant therapy and lifestyle factors should be considered. The identification and validation of genetic biomarkers of TNF-i response in PsA, would enable a stratified medicine approach to be adopted clinically, reducing economic burden and improving quality of life.

## Data Availability

The data that support the findings of this study are available on request from the corresponding author, JB. The data are not publicly available due to privacy/ethical restrictions.
